# Determination of Morphogenetic and Diurnal Variability in Phenolic and Flavonoid Content of *Echinacea purpurea* (L.) Moench: A Potential Source of Natural Anioxidants

**DOI:** 10.1007/s11130-025-01315-w

**Published:** 2025-03-10

**Authors:** Bilge Ozcan, Nejdet Sen, Mustafa Resul Demiray, Ibrahim Bulduk, Ercument Osman Sarihan, Mehmet Ugur Yildirim

**Affiliations:** 1 Faculty of Medicine, Department of Pharmacology, Uşak University, Uşak, 64200 Turkey; 2 Department of Chemical Engineering, Selçuk University, Konya, 42130 Turkey; 3 Faculty of Engineering, Department of Chemical Engineering, Afyon Kocatepe University, Afyonkarahisar, 03200 Turkey; 4 Faculty of Agriculture, Department of Field Crops, Uşak University, Uşak, 64200 Turkey

**Keywords:** Bioperiodicity, Caffeic acid, Chlorogenic acid, Diurnal Rhythm, *Echinacea*, Phenolic Acid, Plant Extracts

## Abstract

*Echinacea purpurea* (L.) Monech has a diverse range of phytochemicals, including flavonoids, polyphenols, phenolic acids, alkylamides, and terpenoids. Phenolic and flavonoid substances derived from the leaves and flowers have been used for treatment of illness. The aim of this study to evaluate the polyphenolic components of the flowers and leaves of *E. purpurea*, along with the diurnal variations and the morphological differences. Two-year-old in full bloom flowers and leaves were harvested at different times of the day (6:00 am, 9:00 am, 12:00 am, 03:00 pm, 06:00 pm, 09:00 pm). The water and methanol extracts prepared from these samples to determine total phenolic, total flavonoid and phenolic compound contents. The experiment was established in randomized complete block design with three factors and three replications. The highest total phenolic content was observed in flowers with methanol extract (157.91 ± 0.32 mg QE/g dry extract) at 09.00 a.m. Also the highest total flavonoid content was observed in leaves with methanol extract at 09.00 a.m (80.12 ± 0.16 mg QE/g dry extract). Chlorogenic acid (19.1 ± 0.83 mg/g methanol extract) and caffeic acid (13.2 ± 0.58 mg/g methanol extract) were identified as the predominant phenolic acids. The peak concentrations of these phenolic acids were observed at 6:00 a.m, followed by a gradual decline throughout the subsequent hours of the day. Findings emphasize the importance of harvesting *E. purpurea* at the optimal time to maximize antioxidant capacity. This study provides valuable new information on morphogenetic and diurnal variation of phenolic and flavonoid content for drug and pharmaceutical raw material production.

**Supplementary Information:**

The online version contains supplementary material available at 10.1007/s11130-025-01315-w.

## Introduction

*Echinacea* is a perennial herbaceous plant that belongs to the Asteraceae family and originates from North America. The *Echinacea* genus comprises 10 species, 11 taxa, and 2 botanical varieties. It can be found across North America and Europe, with the most frequently utilized species in traditional medicine being *Echinacea purpurea* (L.) Moench and *Echinacea pallida* Nutt [1]. The roots and aerial parts have been traditionally used by Native Americans to treat wounds, burns, eczema, insect bites, snake bites, toothaches, throat infections, pain, coughs, tuberculosis, and stomach cramps [2]. Additionally, it has been empirically demonstrated to exhibit therapeutic properties, encompassing antioxidant, anti-inflammatory, antitumor, antimicrobial, immunomodulatory, antidiabetic, hepatoprotective, neuroprotective, antifungal, antiviral, gastrointestinal protective, and cannabimimetic activities [3]. It possesses a diverse range of phytochemicals, including flavonoids, polyphenols, phenolic acids, alkylamides, polysaccharides, glycoproteins, volatile oils, fatty acids, aldehydes, and terpenoids. The main phenolic acids include caffeic acid, chlorogenic acid, cichoric acid, caftaric acid, and echinacoside [3, 4]. The antioxidant efficacy of plants is associated with their chemical composition, primarily attributable to elevated levels of total phenolic content, total flavonoid content, terpenoids, alkaloids, and organic acids. Previous studies have indicated that various parts of the *Echinacea* plant—roots, stems, leaves, and flowers—harbor differing concentrations of polyphenols and phenolic acids and exhibit varied levels of antioxidant activity [5, 6].

Given their impact on health, it is crucial to obtain natural antioxidants with high effectiveness through efficient techniques and to assess their antioxidant properties accurately. The composition and nature of bioactive compounds, particularly phenolics, can exhibit considerable variations based on distinct plant components (leaves, flowers, roots, or stems), processing parameters (extraction techniques, storage conditions, etc.), as well as environmental and agricultural factors (cultivation conditions, harvest season, and timing) [7]. Importantly, in addition to these the diurnal cycle has a significant impact on various metabolic and physiological functions in plants. During 24 h a day, temperature, humidity, light quality, and intensity all fluctuate. These elements directly influence nutrient absorption and the process of photosynthesis, affecting the generation and processing of secondary metabolites like phenols, flavonoids, phenolic acids, and essential oils [8, 9]. Research has indicated that the variabilty of secondary metabolites in the same plant increases at different hours [10].

The existing literature demonstrating the diurnal variations in the phenolic and flavonoid profiles of *E. purpurea* is significantly lacking. The objective of this research was to fill the knowledge gap in the literature by evaluating the diurnal variation in the phenolic content of the flowers and leaves of *E. purpurea.* (L.) Moench.

## Materials and methods

This section is presented as supplementary material Sects. [11–14].

## Results and Discussion

### Total Phenolic Contents

The diurnal variation of total phenolic content (TPC) in flowers and leaves is given in Table 1.

**Table 1:** Diurnal variability in total phenolic content in *E. purpurea* flowers and leaves mean ± SD (mg GA/g dry extract)

	Harvest Time	Flowers		Leaves	
		Methanol	Water	Methanol	Water
Total Phenolic Substance (mg GA/g dry extract)	06:00 am	109.76 ± 0.23	104.67 ± 0.22	56.49 ± 0.12	53.82 ± 0.11
	09:00 am	157.91 ± 0.32	100.46 ± 0.20	65.38 ± 0.13	84.32 ± 0.17
	12:00 am	113.57 ± 0.24	99.32 ± 0.19	76.34 ± 0.15	52.45 ± 0.10
	03:00 pm	85.35 ± 0.17	103.73 ± 0.21	52.15 ± 0.11	110.35 ± 0.22
	06:00 pm	91.43 ± 0.19	91.90 ± 0.18	44.45 ± 0.09	86.92 ± 0.17
	09:00 pm	88.65 ± 0.18	120.36 ± 0.24	68.74 ± 0.14	114.81 ± 0.23

The differences between triple, double, and general means were found to be statistically significant at the *p* < 0.01 level in the studies of the impact of three factors (Plant part x Solvent x Harvesting time) on the TPC (Table 2).

**Table 2:**
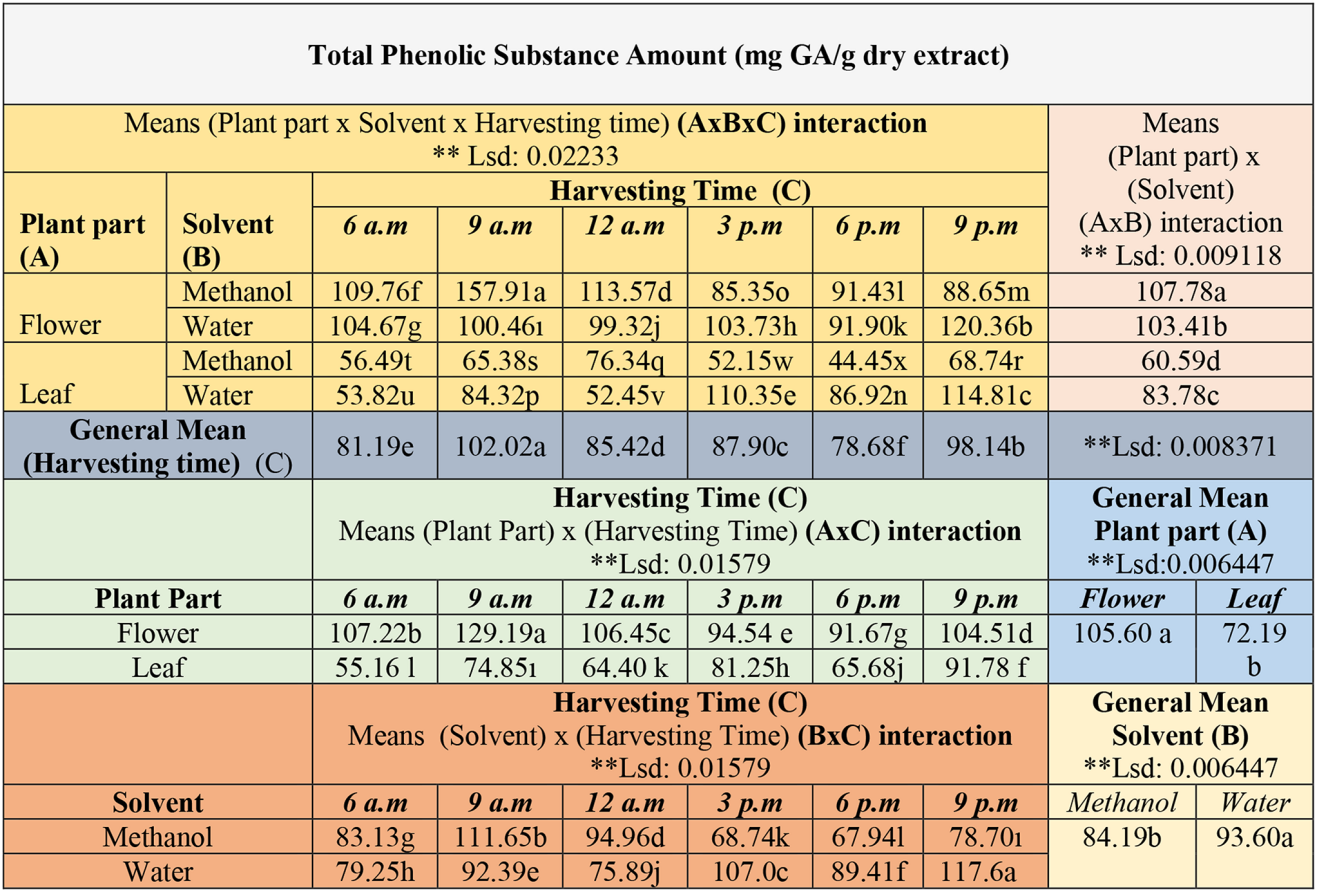
Comparison of diurnal variability in total phenolic content in *E. Purpurea* (AxBxC, AxB, AxC, BxC interactions, general means of plant parts, solvent, harvesting time)

**significant value p<0.01. The differences between the mean values in the tables, shown in different colors, are indicated by letters

In the general mean values of the triple interaction analysis, the highest TPC was 157.91 mg GA/g from flowers in methanol extraction and harvested at 9 am. The subsequent highest values were recorded in the water extract: 120.36 mg GA/g from flowers and 114.81 mg GA/g from leaves at 9 pm. According to the findings, flowers showed higher concentration compared to leaves (*p* < 0.01, Table 2, AxBxC interaction). Regarding the general mean of plant parts, the flowers have contained 105.60 mg GA/g, while the leaves contained 72.19 mg GA/g. The flower parts were therefore richer and had a total of 33.41 mg GA/g higher TPC (*p* < 0.01, AxC interaction). Previous studies also showed that TPC is higher in flowers than in leaves [5, 6, 15]. Lin et al. reported that flowers contain more TPC than leaves, stems, and roots, with a peak measurement of 17.35 mg GA /g [5]. Additionally, another study showed that both phenolics and flavonoids peaked during the flowering phase; TPC was 53.29–78.34 mg GA /g extract [6]. Compared to the general mean of the solvents, the highest quantity was derived from water extraction, 93.60 mg GA/g, followed by methanol extraction, 84.19 mg GA/g (*p* < 0.01, BxC interaction). Water extraction will yield the highest efficiency in phenolic compounds by selecting flowers from the segments of the plant designated for use as drog. Furthermore, water offers advantages in numerous areas compared to methanol; it is more beneficial to health, more environmentally sustainable, and more cost-effective [16].

**Fig. 1:**
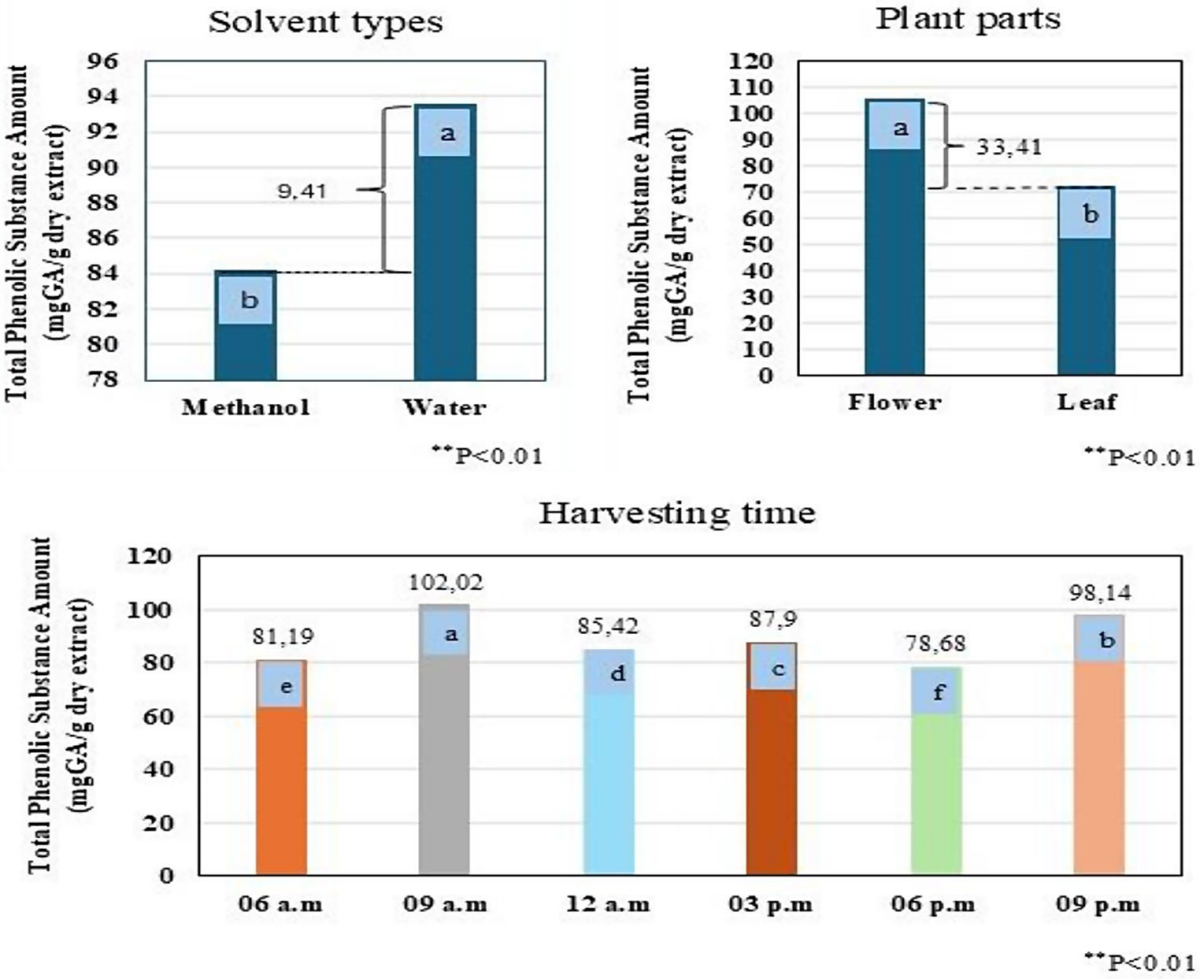
Diurnal variability of the total phenolic content according to solvent types, plant parts and harvesting time in *E. purpurea* (mg GA/g dry extract). The letter(s) on each column indicate significance at *p* < 0.01

Regarding the general mean for harvest times, the maximum compound was observed in plants collected 102.02 mg GA/g at 9:00 a.m., with the next highest being those collected 98.14 mg GA/g at 9:00 p.m. (Table 2, Harvesting Time (C)). These findings suggest that the plant’s concentration of phenolic content is higher after sunrise in the morning and at sunset in the evening (Fig. 1). Moreover, the morning and evening periods for harvesting, during which phenolic compounds are more effectively extracted in water extraction, will enhance the quality of the drog to a greater extent concerning.

## Total Flavonoid Content

The diurnal variation of total flavonoid content (TFC) in flowers and leaves is given in Table 3.

**Table 3:** Diurnal variability in total flavonoid content in *E. purpurea* flowers and leaves mean ± SD (mg QE/g dry extract)

	Harvest Time	Flowers		Leaves	
		Methanol Extract	Water Extract	Methanol Extract	Water Extract
Total Flavonoid Substance Amount (mg QE/g dry extract)	06:00 am	26.47 ± 0.05	12.31 ± 0.03	70.52 ± 0.14	9.18 ± 0.02
	09:00 am	9.95 ± 0.02	9.39 ± 0.02	80.12 ± 0.16	4.82 ± 0.01
	12:00 am	13.81 ± 0.03	6.41 ± 0.01	77.71 ± 0.15	23.09 ± 0.05
	03:00 pm	18.23 ± 0.04	7.46 ± 0.01	25.42 ± 0.05	5.20 ± 0.01
	06:00 pm	6.84 ± 0.01	30.25 ± 0.06	48.23 ± 0.09	3.66 ± 0.01
	09:00 pm	16.41 ± 0.03	45.00 ± 0.09	69.01 ± 0.13	4.60 ± 0.01

The differences between triple, double, and general means were found to be statistically significant at the *p* < 0.01 level in the studies of the impact of three factors (Plant part x Solvent x Harvesting time) on the TFC (Table 4).

**Table 4:**
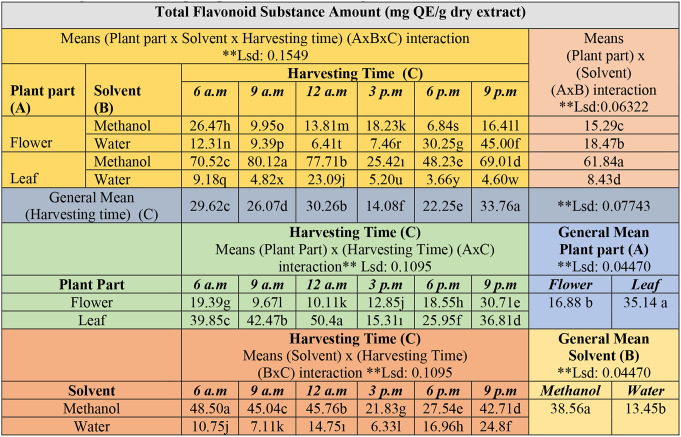
Comparison of diurnal variability in total flavonoid contents in *E. Purpurea* (AxBxC, AxB, AxC, BxC interactions, general means of plant parts, solvent, harvesting time)

**significant value p<0.01. The differences between the mean values in the tables, shown in different colors, are indicated by letters

In the general mean values of the triple interaction (plant part x solvent x harvest time), the highest quantity of flavonoid content, 80.12 mg QE/g, was obtained from leaves using methanol extraction which harvested at 9 a.m. Also the subsequent highest value was recorded 77.71 mg QE/g from leaves in methanol extract at 12 p.m. The results showed that leaves had a higher concentration of TFC compared to flower (*p* < 0.01, Table 4, AxBxC interaction). In relation to the general mean of plant parts, the leaves have contained 35.14 mg QE/g, while the flowers contained 16.88 mg QE/g. The leaf parts were therefore richer, having 18.26 mg QE/g more flavonoid content in total (*p* < 0.01, AxC interaction). Previous studies indicated that TFC during the flowering period was higher than during the greening, budding, and seed maturation periods, and determined to be 32.09–47.98 mg QE/g [6]. In another study, whole plant extracts had obtained a TFC of 32.3 mg GA/g by classical extraction techniques, which was higher than 27.0 mg GA/g from ultrasound extraction. Also, TPC was recorded as 60.2 ± 0.1 mg GA/g via classical extraction and 46.8 ± 0.3 mg GA/g with ultrasound extraction [17]. In further research focusing on whole plant extracts, the TFC was determined to be 86.0 ± 4.6 mg QE/g extract, while it uniquely exhibited lower total phenolic levels when compared to flavonoids, measuring 22.3 ± 1.0 mg GAE/g [18]. Contrary to previous research, this study found that the flavonoid levels in the leaves surpassed that of the flowers. Additionally, the leaves had concentrations ranging from 25.42 ± 0.05 to 80.12 ± 0.16 mg QE/g in methanol extract.

**Fig. 2:**
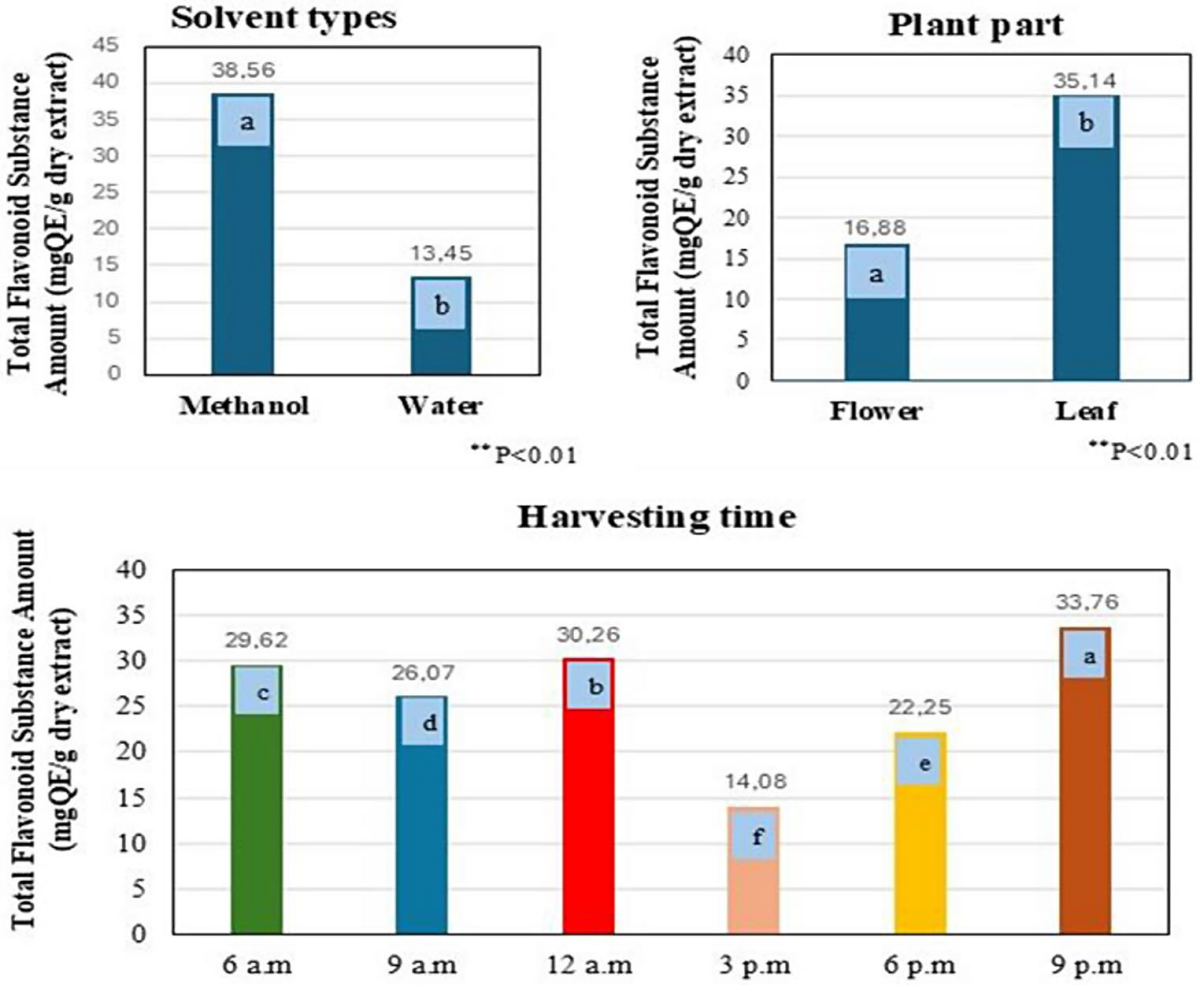
Diurnal variability of the total flavonoid content according to solvent types, plant parts and harvesting time in *E. purpurea* (mg QE/g dry extract). The letter(s) on each column indicate significance at *p* < 0.01

Compared to the general mean of the solvents, the highest quantity was derived from methanol extraction 38.56 mg QE/g, followed by water extraction 13.45 mg QE/g. Methanol had extracted more flavonoid compounds and contained a total of 25.11 mg QE/g more in total (*p* < 0.01, BxC interaction). The daily variations of TFC in methanol extracts from the leaves consistently yielded higher amounts than those from the flowers throughout all hours. Regarding the general means for harvest times, the highest amount of TFC was obtained at 9:00 p.m. (33.76 mg QE/g), followed by at 12:00 p.m. (30.26 mg QE/g) and at 6:00 a.m. (29.62 mg QE/g). These results indicate that the accumulation of flavonoid compounds is higher during sunset in the evening (*p* < 0.01, Harvest Time (C)). Harvesting the leaf parts during sunset or from morning to midday will achieve the optimum yield and improved quality of the drog and extracting them with methanol if the objective is to collect flavonoids (Fig. 2). Other studies on plants have indicated that the levels of phenolic compounds exhibit diurnal variations; for instance, *Hypericum* species peaking at noon [19], *Brassica* species broccoli and cabbage accumulate the most phenolics during the nighttime hours before dawn, while Chinese cabbage and turnip greens achieve their highest levels of accumulation at dawn [20]. Also, flavonoids have fluctuations in daily rhythm in plants like ferns (*Cryptogramma crispa)*, grape berries *(Vitis vinifera)*, tropical trees (A*nacardium excelsum)*,* and* strawberry fruit (*Fragaria x ananassa*) [8]. Since the synthesis of secondary metabolites in each plant varies at specific hours unique to that species, it is essential to conduct precise analyses tailored to each plant. For *E. purpurea* viewed from an agricultural standpoint, it is projected that harvesting in the morning and during sunset hours will yield high amounts of both compounds. Due to the similarity of harvesting times, both flavonoid and phenolic compounds will be collected and separated simultaneously, providing phenolic content to the collected flowers and flavonoid content to the leaves, thus enhancing the quality of the relevant compounds for drog extraction with appropriate solvents. Furthermore, conducting the harvesting process in high temperatures during the day will impair worker performance. Harvesting in the early morning and evening is a more cost-effective and efficient choice that will improve productivity.

## Phenolic Compound

**Fig. 3:**
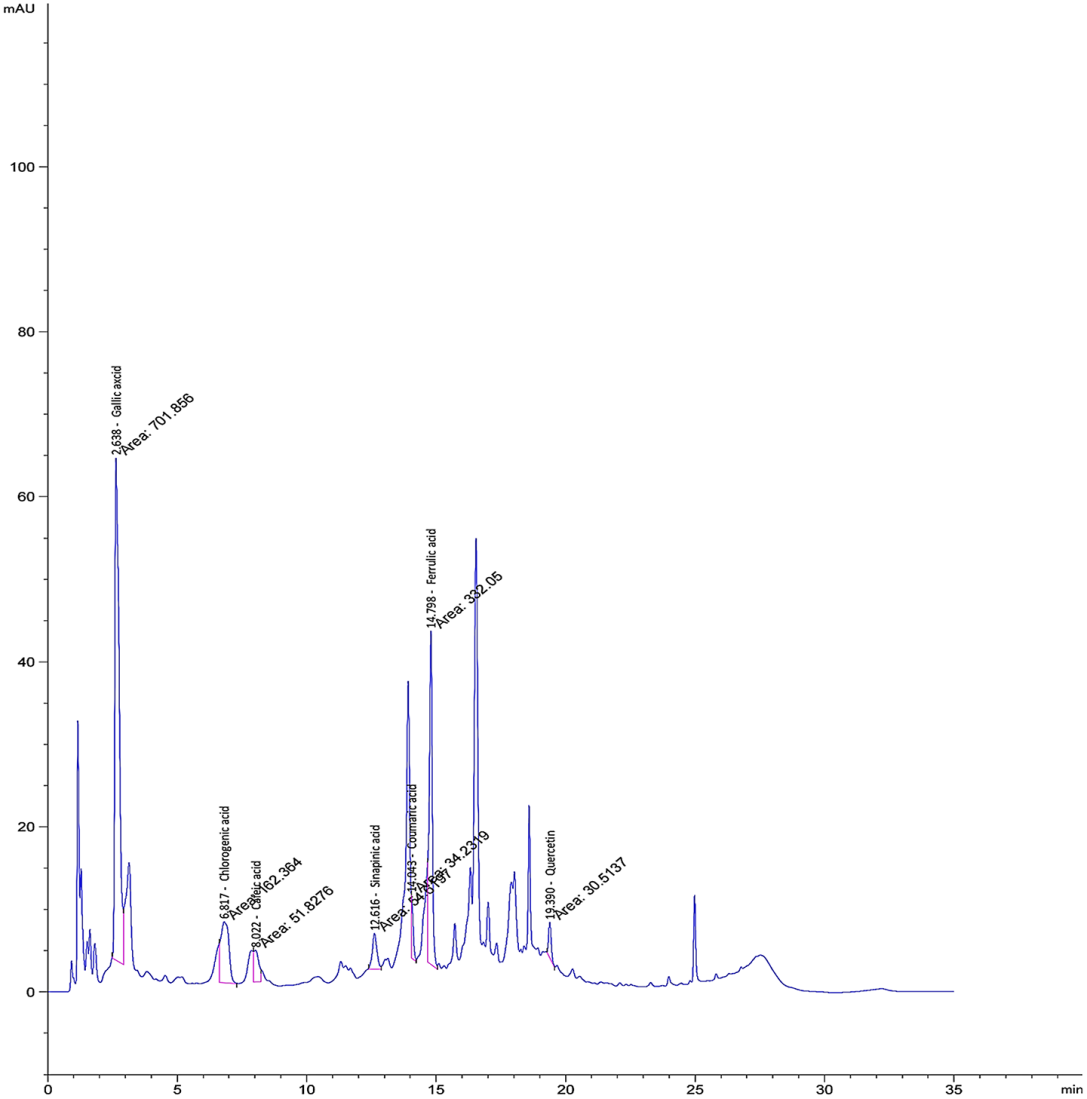
The chromatogram of the sample solution

In the HPLC analysis conducted for phenolic compounds, quercetin, chlorogenic acid, caffeic acid, gallic acid, coumaric acid, protocatechuic acid, ellagic acid, ferulic acid, and syringic acid were detected (Fig. 3).

The quantitative analysis of phenolic compounds in leaves and flowers are given in Table 5.

**Table 5:**
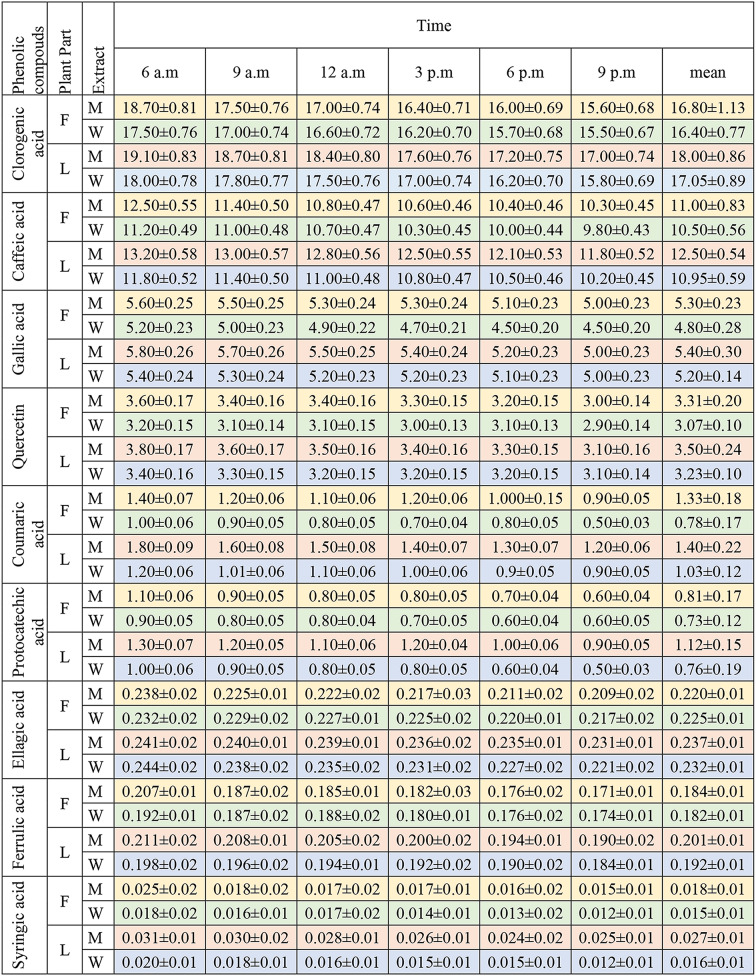
The profile of phenolic acids in flowers and leaves of coneflower in methanol and pure water extract mean ± SD (mg/g dry extract). F: Flower, L: Leaf, M: methanol, W: water

The analysis revealed that the concentrations of phenolic acids in the leaves were higher than in the flowers at all times, peaking at 6 a.m. and subsequently declining steadily during the other hours to reach their lowest point by 9 pm. Furthermore, the values in the methanol extract were higher than in the water extract. In addition, chlorogenic acid and caffeic acid were at the highest concentrations in both flowers and leaves among phenolic acids (Table 5). In earlier research, chlorogenic acid and caffeic acid were mainly found in the flowers, followed by the roots, leaves, and stems sequentially. The amount of chlorogenic acid was 0.22 mg/g, while caffeic acid was present at 0.05 mg/g [5]. In an another study, chlorogenic acid 2.81 ± 0.23 mg/g and caffeic acid 0.40 ± 0.03 mg/g were recorded in flowers [21]. Differently, Chen et al. showed that chlorogenic acid levels in leaves were 5.96 ± 3.76 mg/g, and in flowers, 0.15 ± 0.19 mg/g [15]. This study showed that the concentration of phenolic acid compounds was greater in the leaves than in the flowers. The highest values recorded were 19.1 ± 0.83 mg/g for chlorogenic acid in the leaves, 18.7 ± 0.81 mg/g in the flowers, 13.2 ± 0.58 mg/g for caffeic acid in the leaves, and 12.5 ± 0.55 mg/g in the flowers. These levels are significantly elevated higher than compared to findings from previous studies. Caffeic acid is also produced in various plants such as olives, coffee beans, berries, potatoes, carrots, fruits, and propolis [22]. It exhibits significant biological activity, including antioxidant [22], anti-inflammatory [23], immunostimulatory, antidiabetic, cardioprotective, antiproliferative, anticancer, hepatoprotective activity, antibacterial, and antiviral activities [24]. The anti-inflammatory effect of caffeic acid involves the inhibition of prostaglandin E2, COX-2, iNOS, and TNF-α expression, as well as a reduction in pro-inflammatory NF-κB signaling [23]. Chlorogenic acid was synthesized through the secondary metabolism of various sources such as tea, coffee, cocoa, and berry fruits [25]. Chlorogenic acid has important biological activities, including antioxidant [26], free radical scavenging, anti-inflammatory [27], antidiabetic, hepatoprotective, cardioprotective, anti-hypertensive, neuroprotective, anti-obesity, anticarcinogenic, antibacterial, and antiviral [25]. Chlorogenic acid exhibits anti-inflammatory and antioxidant activity by reducing NF-κB, LPS-induced TLR-4, and TNF signaling, while suppressing caspase activities. Chlorogenic acid has been shown to decrease autophagy and apoptosis by lowering the levels of MAP1LC3B, BAX, TP53, and CASP3 [25]. The significant amounts of chlorogenic and caffeic acids derived from the plants in this study suggest that these plants can be effectively used the aforementioned effects. Extrinsic and intrinsic factors influence the complex synthesis and accumulation of secondary metabolites in medicinal plants. These factors, such as the plant’s genetics [28], region’s climate, temperature, humidity, soil composition, timing of collection [29, 30], drying techniques, and the storage conditions [31], as well as the extraction solvents and the denaturation from the temperatures achieved during the extraction process, influence the extraction and quantity of these compounds [32]. All these parameters are crucial for determining and implementing the successful extraction and accurate evaluation of secondary metabolites from food and medicinal plants. This study indicated that the timing of plant collection should align with the selected extraction solvent to achieve maximal efficiency.

## Conclusion

This research, for the first time, revealed the daily variations of total phenolic content, total flavonoid content, and phenolic compounds in *E. purpurea.* Moreover, it revealed that the type of solvent and the part of the plant greatly affect the total phenolic and flavonoid levels. The flowers contained the highest TPC, and water extraction provided the most effective results as a solvent. Harvesting in the morning, around 9:00 am, yielded the best results for total phenolic content. In contrast, the leaves had the highest TFC, and methanol extraction yielded the most effective results as a solvent, with the optimal harvesting time being in the evening, around 9:00 p.m. Additionally, phenolic acids exhibited distinct daily concentration patterns, peaking in the early morning. These findings emphasize the importance of harvesting *E. purpurea* at the optimal time to maximize antioxidant content. It is crucial to acquire a natural active compound with specific efficacy and quality for treatment use. Consequently, further research is required on the ecological, ontogenetic, and morphogenetic factors that influence the variation of secondary metabolites.

## Electronic Supplementary Material

Below is the link to the electronic supplementary material.

Supplementary Material 1

## Data Availability

No datasets were generated or analysed during the current study.
