# Supplementary material for: Determination of Morphogenetic and Diurnal Variability in Phenolic and Flavonoid Content of Echinacea purpurea (L.) Moench: A Potential Source of Natural Anioxidants
Source: Plant Foods Hum Nutr. 2025 Mar 10;80(1):88. doi: 10.1007/s11130-025-01315-w (PMC11893669; doi:10.1007/s11130-025-01315-w)
Supplement: Supplementary file 1 — Supplementary Material 1 [file 11130_2025_1315_MOESM1_ESM.docx]

**Supplementary Material**

**Determination of Morphogenetic and Diurnal Variability in Phenolic and Flavonoid Content of *Echinacea purpurea* (L.) Moench: A Potential Source of Natural Anioxidants**

**Bilge OZCAN^1^, Nejdet SEN^2^, Mustafa Resul DEMIRAY^2^, Ibrahim BULDUK^3^, Ercument Osman SARIHAN^4^, Mehmet Ugur YILDIRIM^4^**

^1^Faculty of Medicine, Department of Pharmacology, Uşak University, Uşak, 64200, Türkiye

^2^Department of Chemical Engineering, Selçuk University, Konya 42130, Türkiye

^3^Faculty of Engineering, Department of Chemical Engineering, Afyon Kocatepe University, Afyonkarahisar 03200, Türkiye

^4^Faculty of Agriculture, Department of Field Crops, Uşak University, Uşak 64200,Türkiye

Correspondence to Bilge OZCAN, [bilge.ozcan@usak.edu.tr](mailto:bilge.ozcan@usak.edu.tr)

**Material and Methods**

**Materials and Sample Collection**

In the study, 2-year-old plants from the *Echinacea purpurea* (L.) Moench. experimental area located in the research and experimental area of ​​Uşak University Faculty of Agriculture were used as material. "Tutar-2012", a registered cultivar of Echinacea purpurea (L.) Moench, was used as material. Plants were obtained from the Aegean Agricultural Research Institute and cultivated in the research field of Uşak University Faculty of Agriculture. The water and methanol extracts prepared from these samples were dried and ground to determine total phenolic, total flavonoid, and phenolic compound contents. Plant harvests were carried out at different times of the day (6:00 a.m, 9:00 a.m; 12:00 a.m; 03:00 p.m; 06:00 p.m; 09:00 p.m) on 22 June, 2022 while the plants were in full blooming stage. Samples were ground after drying. It was prepared for analysis to determine the changes in phenolic compounds during the day.

**Chemical Substances**

Quercetin (QE), gallic acid (GA), sodium acetate trihydrate, aluminum chloride (Sigma-Aldrich Chemie GmbH), and methanol (Merck).

**Plants Extraction**

Dried and powdered plants were extracted using the Soxhlet apparatus using the continuous extraction method. The dry test sample (plant powder) was placed in a cartridge made of ordinary filter paper and placed in the soxhlet apparatus. Each sample was extracted with two different solvents, pure water and methanol, for approximately 4 hours. The solvent was evaporated in the evaporator. Then it was left to dry in the oven at 45ºC.

**Determination of Total Polyphenols**

The total phenol content in the extracts was determined according to the Folin-Ciocaltaeu method [S1]. Some changes were made while applying the method. Solutions of pure water and methanol extracts were prepared using methanol to be 500 ppm. Gallic acid standard, which is a phenolic acid, was used to prepare the standard graph. Gallic acid standard solutions were prepared in methanol. Solutions of gallic acid at different concentrations were prepared (1000, 500, 250, 125, 62.5, 31.25, 15.625, 7.8125 ppm). A standard working graph was prepared by showing the absorbance values ​​at 765 nm on the y-axis and the concentration values ​​corresponding to the absorbance on the x-axis. The R^2^ value of the graph was determined as 0.9999 (Fig. S1). This value is of great importance in terms of the sensitivity of the study. According to the graph, the total phenolic substance in the plant extracts was calculated as mgGA/g dry extract and plotted. 0.1 mL of the plant extract solutions prepared as 500 mg/L was taken, 7.9 mL of pure water, 0.5 mL of Folin-Ciocalteu reagent and finally 20% sodium carbonate solution were added and mixed and incubated at 40°C for 30 minutes. At the end of the period, it was measured in a UV-Vis Spectrophotometer device at a wavelength of 765 nm. The same procedures were applied to gallic acid solutions prepared at different concentrations to draw the calibration graph. Methanol was used instead of the plant sample solution as a blank.

**Fig. S1** Gallic acid calibration graph, prepared for total phenolic substance determination.

**Determination of Total Flavonoids:**

To determine the total flavonoid amount, the aluminum chloride (AlCl_3_) colorimetric method was applied for the total flavonoid content of plant extracts developed by Woisky and Salatino[S2]. Solutions of pure water and methanol extracts were prepared using methanol to be 500 ppm. Quercetin (QE) was used to prepare the standard graph. Quercetin standard solutions were prepared with methanol. Solutions of quercetin at different concentrations were prepared (600, 400, 200, 100, 50, 25 ppm). A standard working graph was prepared by showing the absorbance values ​​at 415 nm on the y-axis and the concentration values ​​corresponding to the absorbance on the x-axis. The R^2^ value of the graph was determined as 0.9997 (Fig. S2). This value is of great importance in terms of the sensitivity of the study. According to the graph, the total flavonoid content in the plant extracts was calculated as mgQE/g dry extract and plotted. 0.5mL of plant extract solutions prepared as 500mg/L were taken and 1.5mL methanol, 0.1mL AlCl_3_, 0.1mL 1M sodium acetate were added and mixed. The mixtures were incubated at room temperature for 30 minutes. At the end of the period, they were read at 415 nm wavelength in the UV-Vis spectrophotometer. The same procedures were applied to quercetin solutions to draw the calibration graph. Methanol was used instead of the plant sample solution as a blank.

**Fig. S2** Quercetin calibration graph, prepared for total flavonoid substance determination.

**Phenolic Compound Analysis Method by HPLC**

The analysis of phenolic compounds in samples was determined by Agilent brand 1260 model HPLC instrument. A column of ACE-C18 (4. 6 mm×150 mm, 5μm) dimensions was employed for the chromatographic separation of phenolic compounds. Mobile phase A consisted of ultrapure water with 0. 1% acetic acid, while mobile phase B was composed of acetonitrile. The flow rate of the mobile phase was maintained at a constant 1. 0 mL min-1. The gradient conditions used in the experiment were as outlined below: from 0 to 3.25 minutes, 8-10% B; from 3.25 to 8 minutes, 10-12% B; from 8 to 15 minutes, 12-25% B; from 15 to 15.8 minutes, 25-30% B; from 15.8 to 25 minutes, 30-90% B; from 25 to 25.4 minutes, 90-100% B; and from 25.4 to 30 minutes, 100% B. The injection volume amounted to 10µL, while the column temperature was upheld at 25 °C. The selection of detection wavelengths was based on the specific wavelengths where phenolic compounds under analysis exhibited peak absorption. Syringic acid, protocatechuic acid, and gallic acid were identified at a wavelength of 280 nm; vanillic acid at 225nm, and p-coumaric acid at 305nm. Caffeic acid and chlorogenic acid were identified at a wavelength of 330nm [S3]. Parameters of the calibration of polyphenolic compound presented in Table S1. 15 different phenolic components; Quercetin, Chlorogenic, Cafeic, Gallic, Coumaric, Protocatechic, Ellagic, Ferrulic, Syringic, Gentisic, Catechic, Vanillic, Sinapinic, Succinic, Carboxylic and Galacturonic acid were analyzed (Fig. S3).

**Table S1** Parameters of the calibration of polyphenolic compound

| No | Compound | Retention  time min. | Equation | Linear range  (µg mL^-1^) | R^2^ | LOD  (µg mL^-1^) | LOQ  (µg mL^-1^) |
| --- | --- | --- | --- | --- | --- | --- | --- |
| 1 | Gallic acid | 2,676±0,010 | y = 51,921x + 50,226 | 5-30 | 0.9999 | 1.30 | 3.90 |
| 2 | Protocatechic acid | 4,328±0,007 | y = 36,149x + 56,761 | 5-30 | 0.9997 | 0.80 | 2.60 |
| 3 | Chlorogenic acid | 6,920±0,005 | y = 27,86x + 26,982 | 5-30 | 0.9996 | 1.00 | 2.90 |
| 4 | Vanilic acid | 7,291±0,004 | y = 31,153x + 74,003 | 5-30 | 0.9999 | 0.80 | 2.40 |
| 5 | Caffeic acid | 7,988±0,004 | y = 42,348x + 42,089 | 5-30 | 0.9998 | 1.10 | 3.30 |
| 6 | Syringic acid | 8,947±0,004 | y = 121,46x + 190,72 | 5-30 | 0.9996 | 0.80 | 2.40 |
| 7 | Sinapinic acid | 12,852±0,003 | y = 50,658x + 120,19 | 5-30 | 0.9995 | 0.80 | 2.40 |
| 8 | Coumaric acid | 14,347±0,003 | y = 216,2x + 209,14 | 5-30 | 0.9996 | 0.90 | 2.90 |
| 9 | Ferrulic acid | 14,620±0,003 | y = 104,94x + 164,78 | 5-30 | 0.9998 | 0.80 | 2.40 |
| 10 | Quercetin | 19,706±0,002 | y = 10,505x + 16,498 | 5-30 | 0.9997 | 0.80 | 2.40 |
| 11 | Ellagic acid | 16,235±0,002 | y = 10,47x + 17,348 | 5-30 | 0,9996 | 1,10 | 3,30 |


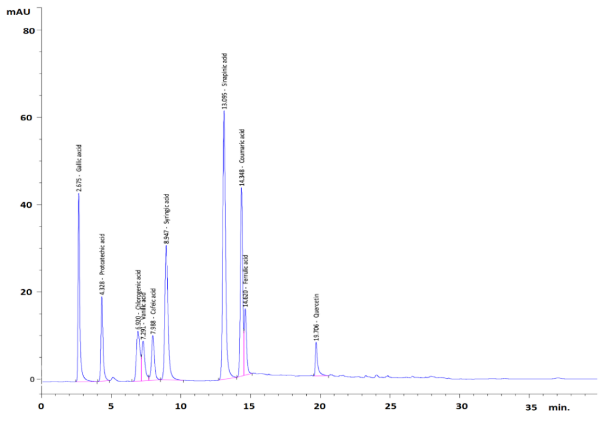


**Fig. S3** The chromatogram of the polyphenol compound standards

**Statistical Analysis**

The experiment was set up with three factors (plant part, solvent, harvest times). The analyses were performed using the MSTAT-C statistical program in accordance with a three-factor completely randomized design. The differences between the average values were determined using the Duncan’s Multiple Range Test [S4].

**Referances**

S1. Gamez-Meza N, Noriega-Rodriguez JA, Medina-Juarez LA, Ortega-Garcia J, Cazarez-Casanova R, Angulo-Guerrero O (1999) Antioxidant activity in soybean oil of extracts from *Thompson grape bagasse*. J Amer Oil Chem Soc 76:1445–1447. <https://doi.org/10.1007/s11746-999-0182-4>

S2. Woisky RG, Salatino A (1998) Analysis of propolis: some parameters and procedures for chemical quality control. J Apic Res 37(10):99–105. <https://doi.org/10.1080/00218839.1998.11100961>

S3. Wen D, Li C, Di H, Liao Y, Liu H (2005) A universal HPLC method for the determination of phenolic acids in compound herbal medicines. J Agric Food Chem 53(17):6624–6629. <https://doi.org/10.1021/jf0511291>

S4. Düzgüneş O, Tahsin Kesici, Orhan Kavuncu, Fikret Gürbüz (1987) Araştırma ve deneme metotları. Ankara Üniversitesi Ziraat Fakültesi Yayınları, vol. 1021.
